# The mediating effect of personal mastery and perceived social support between emotional intelligence and social alienation among patients receiving peritoneal dialysis

**DOI:** 10.3389/fpubh.2024.1392224

**Published:** 2024-06-13

**Authors:** Keke Diao, Jiajia Wang, Yanjun Zhang, Yijia Huang, Yan Shan

**Affiliations:** ^1^The Third Affiliated Hospital of Zhengzhou University, Zhengzhou, China; ^2^School of Nursing and Health, Zhengzhou University, Zhengzhou, China

**Keywords:** peritoneal dialysis, emotional intelligence, personal mastery, perceived social support, social alienation

## Abstract

**Aim:**

This study aims to assess the extent of social alienation in patients undergoing peritoneal dialysis and examine how personal mastery and perceived social support mediate the association between emotional intelligence and social alienation in this patient population.

**Methods:**

This study adopts a cross-sectional survey design. A total of 192 patients were recruited from a tertiary hospital located in Henan Province, China, using a convenience sampling method. We have developed a structural equation model to investigate the mediating influence of personal mastery and perceived social support on the emotional intelligence and social alienation of patients undergoing Peritoneal dialysis.

**Results:**

Peritoneal patients exhibited an social alienation score of 42.01 ± 3.15. Elevated EI levels (coefficient = −0.616, *p* < 0.001) were significantly correlated with reduced social alienation. The mediation model demonstrated that personal mastery and perceived social support fully mediated the impact of emotional intelligence on social alienation.

**Conclusion:**

The social alienation of peritoneal dialysis patients is serious, and healthcare professionals should pay attention to patients’ social alienation, improve patients’ emotional intelligence through relevant interventions, increase personal mastery and perceived social support, and finally reduce social alienation.

## Introduction

Chronic kidney disease (CKD) is a worldwide public health concern, affecting approximately 13.4% of the global population (1). Patients with chronic kidney disease face a threefold higher risk of heart failure and an increased susceptibility to urinary tract infections, gastrointestinal bleeding, hip fractures, and other related comorbidities (2). These complications significantly diminish patients’ quality of life. Progressive deterioration of kidney function ultimately leads to the development of end-stage renal disease (ESRD), necessitating renal replacement therapy for survival. Research indicates a projected increase in the number of patients requiring renal replacement therapy, expected to rise from 2,455,000 in 2016 to 5,439,000 by 2030 (3).

Peritoneal dialysis, as a modality of renal replacement therapy, has gained acceptance among patients and is utilized in various countries and regions due to its attributes, including the preservation of residual renal function, the option for home-based administration, and cost-effectiveness (4). Globally, 10–20% of patients are currently undergoing peritoneal dialysis treatment (5). Continuous Ambulatory Peritoneal Dialysis (CAPD) refers to patients exchanging dialysate 3–5 times per day, with each exchange volume of 1.5–2 L. Dialysate is left in the peritoneal cavity for 4–6 h/time during the daytime, and 10–12 h during the night. CAPD is the most dominant peritoneal dialysis modality, accounting for 90% of PD patients (6).

Regrettably, Dialysis fluid exchange at specific times and places restricts patients’ travel and the irreversible aggravation of their psychological burden by their kidneys, leading to the prominence of the phenomenon of “social alienation” (SA), such as peritoneal dialysis patients’ active avoidance of social interaction and the feeling of being alienated by others (7). SA is an objective phenomenon in which an individual is detached from social relationships, accompanied by emotional experiences such as loneliness and indifference, as well as avoidance and rejection of social activities (8). Qing-Er et al. (9) conducted a questionnaire survey involving 300 peritoneal dialysis patients, revealing that 70% of them exhibited deficits in social functioning. Tao Weiwei et al. (10) discovered that, following dialysis, 36.3% of patients experienced reduced interaction with family and community members, rarely engaging in group activities. Moreover, 14.6% of patients remained entirely homebound, with this figure escalating to 84.93% in rural regions. Cuevas-Budhart et al. (11) conducted in-depth interviews with 29 peritoneal dialysis patients across 13 hospitals in Mexico. Their findings indicated that patients’ experiences of family isolation and socioeconomic difficulties led to somatization of emotions, resulting in manifestations of sadness, anxiety, loneliness, and social abandonment.

The SA threatens peritoneal dialysis patients’ physical and mental health. Research has demonstrated that SA induces hypofunction of the hypothalamic–pituitary–adrenal (HPA) axis, subjecting patients to a pro-inflammatory milieu, thus exacerbating the inflammatory aspects of diseases like atherosclerosis, thereby becoming a significant risk factor for cardiovascular disease (11, 12). Furthermore, severe SA exhibits a substantial association with rapid renal failure in patients, serving as a prognostic indicator for the deterioration of renal function and the acceleration of disease progression (13).

Emotional intelligence (EI) is the ability of an individual to recognize and control his or her own emotions and those of others, and to be able to use emotions correctly to deal with and solve problems (14). Previous studies have found a correlation between EI and SA (15), enhancing emotional intelligence and promoting positive emotional expression in patients can help expand social networks and restore social functioning (16). However, how EI affects SA remains a black box that needs to be explored in further research. Understanding the mechanisms of influence will help healthcare professionals to develop targeted interventions to alleviate social alienation in peritoneal dialysis patients.

According to the Cascading Model, the association between EI and behavioral performance is not straightforward; instead, it is influenced by intervening variables (17). Consequently, it is reasonable to hypothesize the presence of mediating variables rather than direct mediators linking EI to SA. The Neurocognitive Processing Model of Emotional Intelligence suggests that emotional intelligence affects social relationships in a way that requires individuals to re-identify and increase their confidence, and that the effectiveness of emotional intelligence relies heavily on reliable feedback from the surrounding environment (18).

Personal mastery is the degree to which an individual perceives control over his or her life and surroundings, and reflects the patient’s self-confidence in life and disease management (19). Furthermore, mentioning the level of EI has been shown to enhance patient self-confidence and elevate self-efficacy, a pivotal factor influencing the personal master (20, 21). It was found that patients with a high personal mastery were more inclined to respond to changes in things with an open and inclusive mindset, actively engage in social interactions, and feel less SA during stressful situations (22, 23). Therefore, this study proposes the hypothesis that EI may improve SA by enhancing patients’ personal mastery.

Perceived social support is an individual perceives or appreciates material or emotional support from the outside world, which reflects the emotional response of the surrounding environment to the patient.

Emotional intelligence was positively correlated with perceived social support, with individuals with high EI subjectively perceiving more social support and utilizing it (24). According to the WHITE heuristic cognitive model of the cognitive theory of emotion, patients in a state of somatic vulnerability tend to actively detach themselves from social activities and persist in negative emotions if the perceived level of social support is low (25). Above all, this study again hypothesizes that EI influences the SA process in which perceived social support plays an important role.

To date, despite some studies examining the connections among EI, personal mastery, perceived social support, and SA, the precise nature of their interrelationships remains ambiguous. Consequently, this study seeks to explore the mediating functions of personal mastery and perceived social support in the relationship between EI and SA among peritoneal dialysis patients.

**Figure 1:**
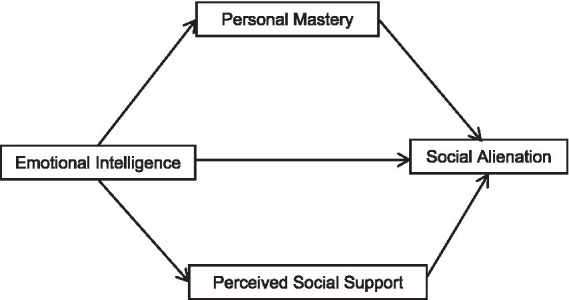
Mediation model.

This endeavor aims to establish a theoretical foundation for intervention strategies aimed at mitigating social alienation within this patient population. Therefore, we developed a mediation model (see Figure 1) to explore the effects and mediating mechanisms of EI on SA in peritoneal dialysis patients, which outlines the hypotheses that (1) EI may affect SA in peritoneal dialysis patients (2) personal mastery and perceived social support mediate EI and SA in peritoneal dialysis patients.

## Methods

### Participants

This study was approved by the author’s university ethics review board and adhered to the principles outlined in the Declaration of Helsinki. This study employed a cross-sectional survey design, and it included peritoneal dialysis patients who met the predetermined inclusion and exclusion criteria. These patients were recruited from the Department of Nephrology at the First Affiliated Hospital of Zhengzhou University between December 2022 and March 2023, utilizing a convenience sampling approach. Inclusion criteria encompassed (a) undergoing peritoneal dialysis more than 1 month; (b) aged ≥18 years; (c) exhibiting stable vital signs; (d) ability to read and understand Chinese language; (e) willing to provide informed consent for active participation in the study.

Exclusion criteria comprised participants who underwent a change in their renal replacement therapy, including transitioning to hemodialysis or receiving a renal transplantation. A total of 202 questionnaires were distributed between December 2022 and March 2023. After eliminating invalid questionnaires, 192 responses were deemed eligible for inclusion.

### Sample size

To ascertain the appropriate sample size, the researcher considered the guideline that the sample size for multiple linear regression analysis should be at least 5–10 times the number of study variables (26). Given the presence of 26 study variables, the sample size was calculated as 7 times this number, resulting in a preliminary sample size of 182. Additionally, to account for potential invalid questionnaires (estimated at 10%), the final sample size was determined to be 202. The study encompassed 202 participants, from whom 192 valid questionnaires were collected, resulting in an effective recovery rate of 95.0%. This high participation rate bolstered the study’s statistical power and ensured the accuracy of its findings.

## Measures

### General information about the patient

We employed a self-administered general information questionnaire to gather pertinent demographic data from the participants. This questionnaire comprised 13 items, encompassing gender, age, marital status, education, occupation, monthly family income (yuan), methods of medical expense payment, living arrangements, time of disease diagnosis (year), duration on dialysis (year), underlying disease etiology, comorbidity count, and biochemical values for calcium, phosphorus, urea nitrogen, and blood creatinine (to be filled out by the researcher after reviewing medical records).

### Wong Law Emotional Intelligence Scale Chinese version (WLEIS-C)

The Wong Law Emotional Intelligence Scale (WLEIS) was developed by Wong and Kenneth in 2002 based on Gross’s theoretical model of emotion regulation (27). Later, Chinese scholars Yifei (28) revised the scale into a Chinese version. The scale comprises four dimensions: self-emotional assessment, emotional assessment of others, emotional control, and emotional use, featuring 16 items. This scale employs a 7-point Likert scale, with responses ranging from “Strongly Disagree” to “Strongly Agree,” yielding a total score between 0 and 96. A higher score indicates a higher level of EI. The WLEIS-C has been validated in a Chinese adult population, and the Cronbach’s alpha coefficient for this scale is 0.89, which is highly reliable.

### General Alienation Scale Chinese version (GAS-C)

The General Alienation Scale (GAS) was developed by Safipour et al. (29) in 1977, and translated and revised into Chinese by Wu Shuang et al. (30). The GAS assesses feelings of alienation concerning personal roles, uncertainty regarding the significance of engagement in activities, and perceived alienation from others. It comprises 15 items organized into four dimensions: alienation from others, skepticism, self-alienation, and meaninglessness. Responses are recorded on a 4-point Likert scale, with each item ranging from 1 to 4, anchored by “strongly disagree” and “strongly agree.” Items 2, 5, and 13 are reverse-scored. The total score ranges from 15 to 60 points, with higher scores indicating a greater severity of SA. The Cronbach’s coefficient for GAS-C applied to peritoneal dialysis patients was 0.902, indicating acceptable reliability (31).

### Personal Mastery Scale Chinese version (PMS-C)

Personal Mastery Scale was developed by Pearlin and Schooler (32), and translated into Chinese by Yu Yibing (33). The PMS assesses an individual’s sense of control over life events’ outcomes. This scale comprises seven items and employs a 5-point Likert scale, with each item scored from 1 (“not at all”) to 5 (“very much”). The total score ranges from 7 to 35 points, with higher scores denoting a stronger perception of individual control. PMS-C has been validated in Chinese peritoneal dialysis patients with reliable reliability (34).

### Perceived Social Support Scale Chinese version (PSSS-C)

Perceived Social Support Scale was developed by Chinese scholars Qianjin (35). The PSSS assesses an individual’s self-awareness and their perceived level of social support. This scale comprises three dimensions: family support, friend support, and support from others, totaling 12 items. Responses are collected on a 7-point Likert scale, with each item spanning from “Strongly Disagree” to “Strongly Agree.” The total score ranges from 12 to 84 points, with higher scores signifying greater perceived social support. A higher score reflects an elevated level of social support comprehension. The scale exhibits strong reliability, as indicated by a Cronbach’s alpha coefficient for this scale applied to Chinese peritoneal dialysis patients of 0.880 (36).

### Statistical analyses

The SPSS 25.0 and Amos 24.0 software were used for data entry and analysis. Continuous variables following a normal distribution were summarized using mean (M) and standard deviation (SD), and differences between groups were assessed via independent samples *t*-tests. Categorical variables were described using counts and percentages, and group differences were evaluated using chi-square (*χ*^2^) tests. Two independent samples t-tests and one-way ANOVA were employed to compare the SA scores of peritoneal dialysis patients with differing characteristics. Multiple stepwise regression analyses were conducted to identify significant predictors of social alienation in peritoneal dialysis patients. Additionally, Pearson correlation analysis was used to examine the relationships between patients’ demographic information, EI, personal mastery, perceived social support, and SA. Multiple linear regression analyses were performed to identify significant predictors of SA in peritoneal dialysis patients. Finally, structural equation models were developed using Amos 24.0, and mediation model testing was carried out utilizing the Bootstrap method, with 2,000 resampling iterations and a two-sided significance level of *α* = 0.05.

The researchers checked the collected data one by one, and entries with more than 20% missing items were deleted. Two researchers entered the data separately and then exchanged the results to ensure that the data were correct.

## Results

### Comparison of SA scores in peritoneal dialysis patients with different characteristics

Among the 192 patients, 98 were male and 94 were female. The mean age of the patients was (42.93 ± 12.64) years, calcium was (2.30 ± 0.39) mmol/L, phosphorus was (2.05 ± 0.52) mmol/L, urea nitrogen was (26.23 ± 6.67) mmol/L, and blood creatinine was (763.66 ± 345.05) μmol/L. Other specific data are shown in Table 1.

**Table 1:** Comparison of social alienation scores in peritoneal dialysis patients with different characteristics (*n* = 192, $\bar{x}$ ±S).

Item	Categories	*N*	%	SA score	*t*/*F*	*p*-value
Age	18-	103	53.6%	43.25 ± 2.74	20.939	<0.001
	45-	76	39.6%	40.51 ± 3.04		
	≥60	13	6.8%	40.92 ± 2.87		
Gender	Male	98	51.0%	41.88 ± 3.32	0.354	0.553
	Female	94	49.0%	42.15 ± 2.98		
Marital status	Unmarried	34	17.7%	43.94 ± 3.23	16.293	<0.001
	Married	118	61.5%	41.08 ± 2.98		
	Widowed or divorced	40	20.8%	43.13 ± 2.47		
Education	Primary school and below	16	8.3%	43.25 ± 2.46	1.688	0.171
	Middle school	68	35.4%	42.28 ± 3.44		
	High school	79	41.1%	41.81 ± 2.71		
	College and above	29	15.1%	41.24 ± 3.75		
Occupation	Unemployed	55	28.6%	42.53 ± 2.43	0.877	0.454
	Full-time work	66	34.4%	41.82 ± 3.67		
	Part-time work	59	30.7%	41.97 ± 3.26		
	Retired	12	6.3%	41.17 ± 2.44		
Monthly family income (yuan)	<1,000	51	26.6%	43.55 ± 2.97	14.481	<0.001
	1,000-	48	25.0%	42.69 ± 2.40		
	3,000-	51	26.6%	41.67 ± 2.78		
	≥5,000	42	21.9%	39.79 ± 3.31		
Living arrangements	Living with spouse	108	56.3%	41.19 ± 2.98	7.963	<0.001
	Living with children	16	8.3%	41.94 ± 2.82		
	Living with parents	29	15.1%	42.62 ± 3.31		
	Living alone	39	20.3%	43.85 ± 2.85		
Time of diagnosis of disease (year)	≤1	25	13.0%	43.92 ± 3.24	6.814	0.001
	1–3	46	24.0%	42.33 ± 3.27		
	>3	121	63.0%	41.50 ± 2.94		
Duration on dialysis (year)	≤1	56	29.2%	44.57 ± 2.54	42.773	<0.001
	1–3	78	40.6%	41.59 ± 2.47		
	>3	58	30.2%	40.10 ± 2.91		
Underlying disease etiology	Glomerular diseases	86	44.8%	41.91 ± 3.34	0.349	0.845
	Diabetic nephropathy	37	19.3%	42.30 ± 2.94		
	Hypertensive nephropathy	32	16.7%	41.75 ± 3.09		
	lupus nephropathy	13	6.8%	42.77 ± 3.44		
	Others	24	12.5%	41.88 ± 2.86		
Comorbidity count	0	62	32.3%	41.77 ± 2.94	0.776	0.509
	1	85	44.3%	42.06 ± 3.37		
	2	27	14.1%	41.74 ± 2.36		
	≥3	18	9.4%	43.00 ± 3.99		

SA, social alienation. The results showed that the SA scores of peritoneal dialysis patients of different age, marital status, monthly family income (yuan), living arrangements, time of diagnosis of disease (year), and duration on dialysis (year) were compared, and the differences were statistically significant (*p* < 0.05).

### Correlation analysis of EI, personal mastery, perceived social support and SA in peritoneal dialysis patients

As shown in Table 2, the total SA score of peritoneal dialysis patients was negatively correlated with the total EI score (*r* = −0.616, *p* < 0.001), with the total Personal Mastery score (*r* = −0.661, *p* < 0.001), and also with the Perceived Social Support score (*r* = −0.696, *p* < 0.001).

**Table 2:** Correlation analysis of EI, personal mastery, perceived social support and SA in peritoneal dialysis patients.

Item	SA total score	Self-alienation	Alienation from others	Skepticism	Meaninglessness
EI total score	−0.616^**^	−0.174^*^	−0.483^**^	−0.422^**^	−0.382^**^
self-emotional assessment	−0.459^**^	−0.117	−0.364^**^	−0.344^**^	−0.256^**^
emotional assessment of others	−0.542^**^	−0.205^**^	−0.425^**^	−0.345^**^	−0.316^**^
emotional control	−0.570^**^	−0.150^*^	−0.427^**^	−0.354^**^	−0.429^**^
emotional use	−0.568^**^	−0.132	−0.459^**^	−0.422^**^	−0.323^**^
Personal mastery total score	−0.661^**^	−0.222^**^	−0.465^**^	−0.397^**^	−0.507^**^
Perceived social support total score	−0.696^**^	−0.214^**^	−0.522^**^	−0.451^**^	−0.474^**^
Family support	−0.546^**^	−0.275^**^	−0.378^**^	−0.265^**^	−0.412^**^
Friend support	−0.590^**^	−0.149^*^	−0.369^**^	−0.499^**^	−0.392^**^
Other people’s support	−0.519^**^	−0.080	−0.480^**^	−0.330^**^	−0.322^**^

^*^p < 0.05, ^**^p < 0.001. EI, emotional intelligence; SA, social alienation.

### Multiple linear regression analyses of SA

The SA score was used as the dependent variable. Age, marital status, monthly family income (yuan), living arrangements, time of diagnosis of disease (year), and duration on dialysis (year), EI, personal mastery, and perceived social support were used as the independent variables, and the values assigned to the independent variables are shown in Table 3. The results showed that age, duration on dialysis (year), EI, personal mastery, and perceived social support were influential factors leading to SA in peritoneal dialysis patients (all *p* < 0.05), explaining a total of 66.1% of the total variance, as detailed in Table 4.

**Table 3:** Assignment of values to independent variables.

Independent variables	Mode of assignment
Age	18–44 = 1, 45–59 = 2,60- = 3
Marital status	Unmarried = 1, Married = 2, Widowed or divorced = 3
Monthly family income (yuan)	<1,000 = 1,1,000- = 2,3,000- = 3, ≥5,000 = 4
Living arrangements	Living with spouse = 1, Living with children = 2, Living with parents = 3, Living alone = 4
Time of diagnosis of disease (year)	≤1 = 1, 1–3 = 2, >3 = 3
Duration on dialysis (year)	≤1 = 1, 1–3 = 2, >3 = 3
EI	Actual scale score
Personal mastery	Actual scale score
Perceived social support	Actual scale score

EI, emotional intelligence; SA, social alienation.

**Table 4:** Multiple linear regression analysis of factors influencing social alienation in peritoneal dialysis patients (*n* = 192).

Independent variable	Regression coefficient	Standard error	Standardized regression coefficient	*t*	*P*-value	VIF
Constant	66.085	1.944		33.997	<0.001	
Age	−0.704	0.241	−0.139	−2.926	0.004	1.286
Marital status	0.161	0.225	0.032	0.718	0.474	1.122
Monthly family income (yuan/month)	−0.107	0.143	−0.038	−0.747	0.456	1.444
Living arrangements	0.021	0.118	0.008	0.181	0.856	1.228
Time of diagnosis of disease (year)	0.282	0.236	0.064	1.194	0.234	1.650
Duration on dialysis (year)	−1.117	0.238	−0.274	−4.702	<0.001	1.939
EI	−0.085	0.019	−0.243	−4.373	<0.001	1.770
Personal mastery	−0.224	0.063	−0.212	−3.553	<0.001	2.041
Perceived social support	−0.161	0.038	−0.272	−4.295	<0.001	2.296

*R*^2^ = 0.682, adjusted *R*^2^ = 0.666, *F* = 44.372, *p* < 0.001. EI, emotional intelligence; SA, social alienation.

### A model test of the relationship between EI, personal mastery, perceived social support and SA in peritoneal dialysis patients

**Figure 2:**
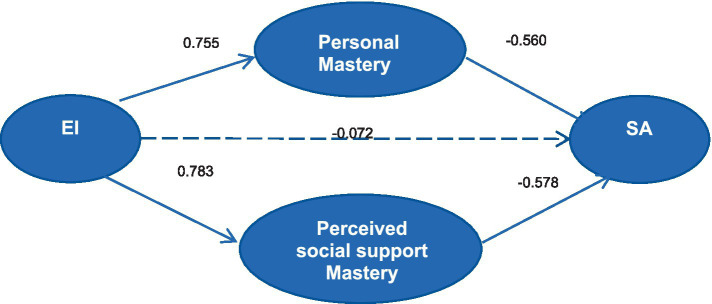
Structural equation modeling of peritoneal dialysis patients’ EI, personal mastery, perceived social support and SA. EI, emotional intelligence; SA, social alienation.

A structural equation model was constructed to test the relationship between EI, Personal Mastery, Perceived Social Support and SA in peritoneal dialysis patients. The model fit indices were calculated using great likelihood estimation: *χ*^2^/df = 1.973(<3), CFI = 0.931(>0.9), GFI = 0. 902(>0.9), IFI = 0.933(>0.9), TLI = 0.909(>0.9), and RMSEA = 0.060(<0.08). The peritoneal dialysis patients’ structural equation models for EI, Personal Mastery, Perceived Social Support and SA are shown in Figure 2.

The results showed that the indirect effect of EI on SA was −0.876, with 95% confidence interval excluding 0, so the indirect effect of EI on SA was established, and since the direct effect of EI on SA was not significant and was not included in the model, the personal mastery and the perceives social support played a fully mediating role in EI and SA at this time. The mediating effect value of personal mastery was −0.423, accounting for 48.29% of the total mediating effect, and the mediating effect value of perceived social support was −0.453, accounting for 51.71% of the total mediating effect. The analysis of effects is shown in Table 5.

**Table 5:** Effect analysis of factors influencing SA in peritoneal dialysis patients.

Effect type	Impact pathways	Estimated value of an effect	LLCL	ULCI	*p*
Direct effect	EI-SA	−0.072	−0.524	0.782	0.736
Indirect effect	EI-personal mastery-SA	−0.423	−0.188	−0.617	<0.001
	EI-perceived social support-SA	−0.453	−0.167	−0.751	<0.001

EI, emotional intelligence; SA, social alienation.

## Discussion

Social alienation (SA) represents an inescapable challenge faced by peritoneal dialysis patients, profoundly impacting their mental well-being. Consequently, it is imperative to scrutinize the determinants of SA and to formulate tailored intervention strategies. In this investigation, the SA score among peritoneal dialysis patients averaged 42.01 ± 3.15, indicating a notably elevated level compared to the scale’s median total score of 37.50. These findings align closely with the scores reported by Xiaofang et al. (37) for lung cancer patients, though they slightly surpass scores observed in stroke patients (38). Several factors contribute to this discrepancy. Firstly, Zhao Cuicui (38) study encompassed a substantial proportion of older adult patients, the cognitive and physical functions of the older adult population are in a stage of decline, which, with increasing age, causes them to be socially inactive and thus aggravates SA. While this study predominantly enrolled young and middle-aged individuals who shoulder diverse family and occupational responsibilities, thus experiencing heightened demands for social engagement (39). Furthermore, the social role of supporting the older adult and caring for children compounds the burdens borne by these patients. Additionally, influenced by traditional Chinese cultural norms, individuals frequently refrain from sharing the impact of their illness with others, believing that such vulnerability does not align with societal expectations. This restraint fosters heightened anxiety, feelings of powerlessness, and a sense of detachment.

Simultaneously, our findings underscore the significance of age and duration on dialysis as pivotal determinants of SA in peritoneal dialysis patients. Surprisingly, our study revealed elevated SA scores among younger patients, a trend contrary to the findings of several previous investigations (40, 41). Notably, dialysis-related complications manifest as discernible alterations in patients’ physical appearance, encompassing generalized edema (42), melanosis (43) and characteristic hyperparathyroidism (44), etc. These conspicuous disease-related attributes are prone to create unfavorable impressions on others, further exacerbated by societal misconceptions and potential instances of social discrimination, thereby inducing psychological distress among patients (44, 45). Younger patients, in particular, grapple with the pronounced impact of these appearance-related changes, which can lead to challenges in pursuing marriage, forming new social connections, and realizing their socialization aspirations (46). Moreover, younger patients often exhibit reticence in sharing their emotions and experiences, ultimately isolating themselves from active engagement in social activities, thus fostering SA (47).

Furthermore, the study results indicate that peritoneal dialysis patients with a dialysis vintage of less than 1 year exhibited higher scores on social alienation, aligning with the findings of Xiaofang et al. (37). Peritoneal dialysis necessitates multiple daily fluid exchanges, meticulous monitoring of fluid balance, and the endurance of complications like pruritus and sleep disturbances (48, 49). For patients with a short dialysis history, striking a swift equilibrium between demanding self-care regimens and their pre-existing work commitments poses a formidable challenge. For patients who have been on peritoneal dialysis for a longer period of time, they have had a rich experience with nephrological care, better adapted to dialysis treatments, and have found the right place in their lives for dialysis, and therefore are less concerned about feeling alienated (50). Consequently, confronted with the stressors of dialysis, these patients often struggle to adapt to and redefine their roles, harboring numerous anxieties and reservations about their treatment. The amplification of psychological symptoms exacerbates their inclination toward social avoidance and SA (51). As a result, healthcare practitioners should direct their attention toward younger patients with shorter dialysis histories, proactively monitoring their emotional shifts. Collaborative interventions, such as patient support associations, can be instrumental in fostering positive emotional experiences, thereby establishing stable social bonds and mitigating their degree of social alienation.

The study’s findings reveal a negative association between higher levels of EI and lower levels of SA among peritoneal dialysis patients. These results align with previous research and contribute to a deeper understanding of the underlying mechanisms (52). Individuals with elevated EI exhibit a propensity to mitigate the impact of stress within their social environment through positive coping strategies, such as cognitive reappraisal. This serves as a protective factor against SA, in accordance with the stress buffer model (53). During this process, individuals tend to refrain from negative emotional expressions (self-containment and avoidance), increase positive emotional experiences, bolster resource development when facing stressful circumstances, and fulfill the prerequisites for social participation. These dynamics align with the extended construction theory (54). It was found that one feature of EI - affective differentiation – neutralizes neural responses to social rejection (i.e., dACC and forebrain insulae) (55), and from this perspective, EI creates conditions for patients to initiate social interactions. These findings lay the groundwork for further exploration of the interplay between personal mastery, perceived social support, EI, and SA among peritoneal dialysis patients.

Our study reveals that personal mastery acts as a complete mediator between EI and SA in peritoneal dialysis patients. According to the theory of limited self-control, self-control resources are finite, allowing individuals only short-term exertion of self-control. Externally stressful situations deplete these resources, diminishing self-control, and potentially resulting in emotional regulation failures (56). Peritoneal dialysis patients, grappling with the enduring burden of lifelong dialysis, job transitions, and life adjustments, often experience a significant erosion of self-worth, which profoundly affects their personal mastery. Moreover, personal mastery is cultivated through continuous social interactions. EI emerges from the amalgamation of self-cognitive processes and emotional management (57). In the realm of emotion regulation and control, patients employ self-beneficial coping strategies, facilitating the transformation of negative emotions into positive ones through cognitive reappraisal. The sustenance of positive emotions fosters self-management confidence, life optimism, and an ensuing personal mastery. Patients possessing high personal mastery exhibit superior disease-related stress coping mechanisms, and through positive outlooks, they confront adverse events related to their condition, thereby fostering improved societal engagement and lower levels of SA (23, 58).

Additionally, our study reveals that perceived social support fully mediates the relationship between EI and SA in peritoneal dialysis patients. Individuals with high EI excel at perceiving and effectively regulating emotions, fostering positive emotional states during social interactions. These positive emotions often extend to family members and friends, fostering positive social interactions, aligning with the concept of emotional contagion (59, 60). Moreover, high EI equips patients to harness valuable social resources effectively. Perceived social support enhances patients’ sense of belonging and identity, compensating for social roles lost due to illness and encouraging proactive engagement in social interactions, ultimately reducing SA (61).

Our study further demonstrates that personal mastery and perceived social support partially mediate the relationship between EI and SA in peritoneal dialysis patients. Notably, navigational social support plays a more substantial mediating role than personal mastery, suggesting that perceived social support may be a stronger predictor of SA. However, additional research is required to validate this hypothesis. This study offers a mechanistic examination of the EI-SA relationship, addressing a gap in previous research. Nevertheless, relevant studies remain limited, and the precise mechanisms require further validation through additional research. Several limitations should be acknowledged. Firstly, this study is cross-sectional, precluding the establishment of causal relationships among variables. Subsequent longitudinal investigations are warranted to validate these findings. Secondly, the study sample was drawn from a single province in China, limiting its generalizability to all peritoneal dialysis patients.

To delve deeper into these intricate connections, future research could explore them through longitudinal surveys and mixed-method studies, incorporating qualitative research components.

## Conclusion

In summary, our study has revealed a pronounced level of SA in peritoneal dialysis patients, with a robust association with EI. Personal mastery and perceived social support emerge as crucial mediators in this relationship. Specifically, implementing an emotional intelligence management program has the potential to enhance patients’ personal mastery and perceived social support, resulting in a subsequent reduction in SA levels. These findings offer a substantive theoretical foundation for addressing SA among peritoneal dialysis patients, suggesting that future clinical practice should consider tailoring interventions, such as the development of an emotional intelligence management program, to augment patients’ personal mastery and perceived social support, ultimately mitigating SA.

## Data availability statement

The raw data supporting the conclusions of this article will be made available by the authors, without undue reservation.

## Ethics statement

The studies involving humans were approved by Zhengzhou University Life Science Ethics Committee. The studies were conducted in accordance with the local legislation and institutional requirements. The participants provided their written informed consent to participate in this study.

## Author contributions

KD: Conceptualization, Methodology, Writing – original draft. JW: Methodology, Writing – review & editing. YZ: Data curation, Methodology, Writing – original draft. YH: Data curation, Investigation, Writing – original draft. YS: Funding acquisition, Resources, Supervision, Writing – review & editing.
